# Correction: *In situ* grown CNTs and electrodeposited MnO_2_ on MXene-carbon nano-fibers for flexible supercapacitors with high energy density

**DOI:** 10.1039/d6cc90067d

**Published:** 2026-02-25

**Authors:** Binhe Feng, Deyang Zhang, Wenbo Guo, Zhaorui Wang, Jinbing Cheng, HaiLong Yan, Tao Peng, Kangwen Qiu, Feng Jing, Yikai Ge, Mengzhen Du, Paul K. Chu, Yongsong Luo

**Affiliations:** a Henan Joint International Research Laboratory of New Energy Storage Technology, Xinyang Normal University Xinyang 464000 P. R. China zdy@xynu.edu.cn; b Henan International Joint Laboratory of MXene Materials Microstructure, Collaborative Innovation Center of Intelligent Explosion-proof Equipment of Henan Province, Nanyang Normal University Nanyang 473061 P. R. China ysluo@xynu.edu.cn; c College of Energy Engineering, Huanghuai University Zhumadian Henan 463000 China; d Department of Physics, Department of Materials Science & Engineering, and Department of Biomedical Engineering, City University of Hong Kong Tat Chee Avenue Kowloon Hong Kong China

## Abstract

Correction for ‘*In situ* grown CNTs and electrodeposited MnO_2_ on MXene-carbon nano-fibers for flexible supercapacitors with high energy density’ by Binhe Feng *et al.*, *Chem. Commun.*, 2026, **62**, 1176–1180, https://doi.org/10.1039/D5CC06218G.

The authors regret that there were errors relating to the ASC device in Fig. 3g and the graphical abstract image of the original article. The revised versions of Fig. 3 (containing Fig. 3g) and the graphical abstract are as shown here.

**Fig. 3:**
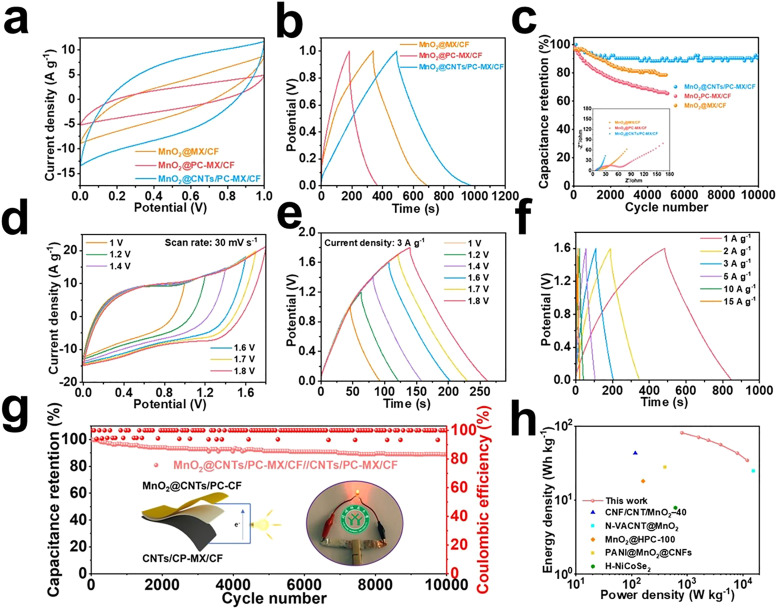
(a) The CV curves of MnO_2_@CNTs/PC-MX/CF, MnO_2_@PC-MX/CF and MnO_2_@MX/CF; (b) the GCD curves of MnO_2_@CNTs/PC-MX/CF, MnO_2_@PC-MX/CF and MnO_2_@MX/CF; (c) cycling performance at 20 A g^−1^ and Nyquist plot; (d) CV curves of ASC at a scanning rate of 30 mV s^−1^; (e) GCD curves of ASC at current density of 3 A g^−1^; (f) GCD curves of ASC at different current densities; (g) cycling stability test of ASC at 10 A g^−1^; (h) Ragone plot.

**Graphical abstract:** 
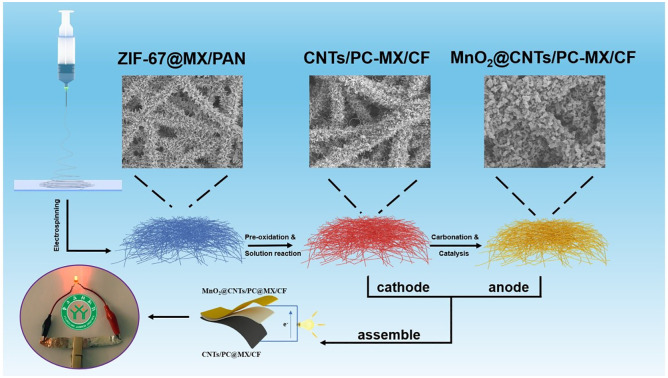


The Royal Society of Chemistry apologises for these errors and any consequent inconvenience to authors and readers.

